# Antiracism in appreciative inquiry: Generative tensions and collective reflexivity

**DOI:** 10.1177/14767503231210418

**Published:** 2023-11-03

**Authors:** Amanda Gebhard, Willow Samara Allen, Fritz Pino

**Affiliations:** Faculty of Social Work, 6846 University of Regina, Canada; Department of Adult EducationFaculty of Education, St. Francis Xavier University, Canada; Faculty of Social Work, 6846 University of Regina, Canada

**Keywords:** Appreciative inquiry, antiracist research, critical qualitative methods, focus groups, immigration and settlement, Covid-19

## Abstract

Appreciative inquiry is an action research methodology focused on revealing an organization’s positive core. As a cross-racial team of antiracist researchers, we were drawn to appreciative inquiry due to its congruences with community-based research perspectives on power-sharing and co-constructing knowledge. Our collaborative reflexivity brought us to question whether Appreciative inquiry’s hyper-focus on positivity would fit our antiracist research paradigm. We articulate reflections of how antiracism theory informed our approach to Appreciative inquiry in a study on the experiences of predominantly racialized settlement workers in schools during the COVID-19 pandemic. We explain how we negotiated tensions between Appreciative inquiry’s focus on positivity and our antiracist framing, in a Canadian settler colonial context where institutional expectations to ignore racism and collapse diversity, loom large. Without a theoretical framework that attends to racism and power, Appreciative inquiry may not fulsomely address participants’ transnational knowledges, nor experiences outside of a positive/negative binary. In our elucidation of how critical reflexivity on racism allowed us to integrate antiracism into Appreciative inquiry, we demonstrate the value of first-person action research for expanding the social justice aims of research.

The seed for our community-based research project was planted at the beginning of the global pandemic. Concerned about the disruptions the pandemic was having on school settlement workers’ ability to serve newcomer^1^ students and families, a non-profit organization for settlement agencies contacted us to establish a research partnership. School settlement workers play multifaceted roles in the lives of newcomer students and their families at critical junctures of integration. The importance of their work was made clear when the pandemic led to the shut down of in-person learning in schools in spring 2020. Yet school settlement workers are an under-researched professional group, who have been paid little attention by school systems and the scholarly community. Attending to this erasure, our research examined how school settlement workers across one Canadian prairie province responded to educational inequities for newcomer students exacerbated by the pandemic. The project was initiated when the pandemic’s disproportionate impacts on racialized newcomers were increasingly visceral, including the exacerbation of racism and xenophobia towards newcomers (Lee & Johnstone, 2021; Lei & Guo, 2022).

We were drawn to appreciative inquiry (Ai) due to its congruences with community-based research perspectives and its orientations to power-sharing and co-constructing knowledge with participants. We were further intrigued by Ai due to critical scholars such as Weiston-Serdan (2017), who posit Ai researchers recognize community organizations “have a host of underrecognized and underappreciated resources that can be built into thriving systems” (p. 68). Similarly, Emery and Flora (2012) contend Ai is suitable for research with communities marginalized by systems of dominance.

As we reviewed the Ai literature and prepared for our focus groups, we questioned whether traditional Ai approaches would be fitting for antiracist research. In particular, we felt a nagging disconcertion with Ai’s seemingly hyper-focus on positivity. Following others who question the positivity aspect of Ai (e.g., Grant & Humphries, 2006), we speculated how positivity as a focal point could silence counternarratives and lived realities of participants. Further, we wondered how Ai risked communicating to participants–a group of predominantly racialized individuals working in a resource-depleted education system during a pandemic-that they should focus on the positive, implying a need for individual, instead of systemic, accountability for change.

How an antiracist theoretical framework shaped our approach to Ai thus became a key discussion topic at every stage of our research, and the central question of this article. Antiracism tenets became the organizing framework for our lines of inquiry and reflexivity. While we report the findings from our study elsewhere (Allen et al., 2021; Gebhard et al., 2021), this methodological paper traces our reflexive process on the methodology of Ai through an antiracist lens. We situate this work within the diverse field of self-reflective, first-person action research approaches (Marshall & Mead, 2005). Marshall’s (1999) reflection about the risk of her research colluding with dominant oppressive structures resonates with us, and the critical reflexivity we detail herein was fuelled by similar concerns. The foregrounding of reflexivity that first-person action research supports (Gearty & Marshall, 2021) was salient for us, because antiracism centers social locations/positionalities and power relations, and demands ongoing and transparent self-reflexivity about how race and other intersecting social locations are always at play.

We begin by describing the socio-political context of this research. We then provide a synthesis of the theoretical framing of antiracism, followed by an explanation of Ai and Critical appreciative inquiry (Cai). We then turn to the insights generated from individual and collective reflection on the tensions of approaching Ai through an antiracist lens. We offer three discussion points: researchers’ critical race consciousness as a requisite for appreciation, revealing the positive core of an organization through a reframing of neoliberal understandings of positivity and happiness, and the recognition of transnational knowledges as requisites for inquiry. We conclude by offering reflections on expanding critical appreciative inquiry scholarship by mobilizing an antiracist approach to inform action-oriented research.

## Context for anti-racism

This research took place in a prairie province in what is known as Canada. The lands have been stewarded since time immemorial by Indigenous peoples^2^ and since being fundamentally disrupted by European invasion and ongoing settler colonization. Under the distinct form of settler colonialism, “invasion is a structure, not an event” (Wolfe, 2006, p. 388), with the ultimate, insatiable goal to claim, clear and exploit lands and resources and to disappear Indigenous peoples (Tuck & Yang, 2012). Today, Indigenous peoples comprise approximately 15% of the province’s population and the remaining population is a diverse group of settlers, who are newcomers and people who have occupied the land for generations. There is extensive discussion elsewhere about non-Indigenous peoples’ relationships with the settler colonial nation-state, Indigenous peoples and Indigenous lands; and on the entanglement of Indigenous dispossession and immigration and settlement processes (see Chatterjee, 2019; Jafri, 2012; Vowel, 2016).^3^

The prairie region is experiencing increasing international immigration (Saskatchewan Bureau of Statistics, 2021). Newcomers continued to settle in Saskatchewan during school closures and for several thousand people, their experience of settlement was almost entirely during the pandemic. The pandemic compounded existing challenges of settlement and integration, including racism (Barker, 2021; Lei & Guo, 2022). In Canada, racism*s* operate to systemically uphold the dominance of white settlers. Despite racial violences, Indigenous peoples, Black people and other racialized people are expected to uphold the narrative that Canada is a place free from structural racial inequality (Thobani, 2007), while bearing the weight of the workload that racism demands of racialized colleagues (Dhamoon, 2020). Such expectations are further layered for racialized immigrants and refugees who are expected to be the ‘grateful’ subjects of Canadian benevolence (Reid, 2021).

In education, racialized newcomer students and families to Canada are marginalized through school cultures, systems, and beliefs that reflect and (over)value white, Eurocentric ways of being and knowing (Nichols et al., 2020). Racism and white settler dominance shape curriculum, teacher practices and attitudes, peer interactions and, consequently, students’ educational trajectories (Gebhard et al., 2022). Settlement workers in schools support newcomer students in navigating oppressive educational landscapes by affirming their multiple identities and ways of knowing, and serving as advocates (Allen et al., 2021).

## Antiracism framework

We employ an integrated antiracism framework that is both a framework for theory and research, and a set of practices that seek to resist and eliminate racism in its interlocking forms (Dei & Adhami, 2022). Antiracism is “an action-oriented educational and political strategy for institutional and systemic change that addresses the issues of racism and the interlocking systems of social oppression” (Dei & Calliste, 2000, p. 13). Consequently, individual awareness of racism (and by association individual ‘privileges’) is insufficient for social and political change (Nath & Allen, 2022). Uprooting structural inequity involves *proactively* resisting oppressive ideologies and practices in all areas of life (Fleras, 2014). Approaches that fixate on celebrating cultural differences and offer inclusions into harmful systems, instead of structural changes, currently proliferate in white-dominant institutions through “the DEI industrial complex” (Gray et al., 2023). Integrated antiracism, rooted in the long-standing activism and scholarship of Black, Indigenous and racialized communities and colleagues, unapologetically reveals how whiteness and power relations sustain racial inequalities within ongoing settler colonial processes (see Thobani, 2021).

## (Critical) appreciative inquiry

Ai began in the 1980s as an action-research methodology grounded in organizational behaviour theory to increase effectiveness in private sectors (Cooperrider & Srivastva, 1987). The methodology emerged from concerns about deficit-based, problem-focused approaches to improving leadership and service delivery in organizations (Hung et al., 2018). Ai in response starts with ‘the positive core’ and ‘what’s working’ to “inspire action for change” (Cockell & McArthur-Blair, 2020, p. 13). Ai has been used in settings including healthcare (e.g., Hung et al., 2018), education (e.g., Evans & Lange, 2019), and community organizations (Emery & Flora, 2012). Researchers have employed Ai to enhance community-based research (De La Ossa, 2005; Paige et al., 2015), and to a more limited extent, to examine racism and activism (Cockell & McArthur-Blair, 2020; Evans & Lange, 2019) and to address diversity and inclusion (Mallory, 2021). Cooperrider and Fry (2020) reflected on the utility of appreciative inquiry for fostering change during challenging times, arguing that “Ai might just reach its highest potential for impact in organizations and human systems in the midst of pandemic, crisis, or tragedy” (p. 268). Critical appreciative inquiry (Cai) brings together social justice, critical theory and appreciative inquiry (Cockell & McArthur-Blair, 2020). Grant and Humphries (2006) were the first to formally articulate the *critical* in appreciative inquiry: “the integrated use of appreciative inquiry and critical theory contributes to deeper insight and recognition of the complexity in human endeavours” (p. 403). Such complexities can be flattened when systemic conditions that (re)produce hegemonic relationships of power and constrain human agency are not addressed (Fitzgerald et al., 2010; Ridley-Duff & Duncan, 2015). Critical Ai recognizes “knowledge-generating capacities are intimately linked to the social system in which they occur...and a person who develops knowledge of the social system will have a variable ability to act, depending on their place within it” (Ridley-Duff & Duncan, 2015, p. 1583).

The narrative multi-phase cycles employed in Ai are flexible (Fifolt & Stowe, 2011), and are interpreted through researchers’ theoretical lenses. Such interpretations extend to the construction of positivity; based on their frameworks, Ai researchers have variable orientations towards what constitutes positivity. A critically reflexive approach suspends the notion that researchers alone should determine what is positive discourse.

## Research process and focus group design

Between November 2020 and January 2021, we conducted eight virtual focus groups with three to five settlement workers in schools (*n* = 30; 25 women and 5 men). Recruitment took place through purposeful sampling, and communications emphasized the voluntary and confidential nature of participation. Participants represented many geographic, racial, ethnic, gender, religious, class and linguistic locations. They spoke languages including Mandarin, Arabic, and Hindi. Participants’ demographic profile included 17 racialized immigrant and newcomer settlers from Asia, South America and the Middle East, and 13 white immigrant and newcomer settlers from Europe, or Euro-Canadian-born white settlers. Participants had backgrounds in education, health, public administration, film/media, and social services. Over half had international professional experience and education, though not all their credentials are recognized in Canada. The majority worked in the province’s largest urban areas (*n* = 22), while eight participants worked in smaller cities and rural communities. Focus groups were recorded, and audio transcripts were open-coded using NVivo software.

Our process was designed around the Five Ds of Ai: Define, Discovery, Dream, Design and Destiny (Fifolt & Stowe, 2011). We took a fluid approach to the 5D cycle, recognizing that to compartmentalize the discussion into specific phases runs contrary to the non-linearity and iterative nature of both storytelling and data analysis. In the Define stage, research questions were established with the advisory group from the partner organization. The next three phases unfolded within the focus groups. In the Discovery phase, *the best of what is*, researchers ask participants about the organization’s positive core. We posed questions about the impact of the pandemic on the strengths of settlement work in schools and the destabilization of previously working support systems for newcomer families. During the Dream phase, *what could be*, researchers encourage participants to articulate a vision for responding to current challenges that align with their strengths. Our questions in this stage encouraged imaginative responses about what supportive, and empowering services for newcomer youth, and how schools *could be* otherwise. In the Design phase, *what should be*, researchers ask participants to share statements of intent that harness the positive core. Guiding questions asked participants to name resources that could enhance their existing wise practice, as well as to articulate what they hoped readers would take away from the research. The Destiny Phase of AI, *what will be*, is accomplished through the formulation of specific recommendations. This final step was actualized in collaboration with our advisory group. We produced a final report of findings and recommendations, held a town hall with community stakeholders, and initiated knowledge dissemination activities and advocacy efforts with settlement agencies and stakeholders (see Gebhard et al., 2021).

Content for this paper was generated through our collaborative reflexive practice. During debriefs after each focus group, we discussed how social locations and power relations shaped what was (un)said about white settler normativity, race and racism; and in turn what knowledge was being (re)produced. These discussions informed our introductory remarks, the questions we each posed, and our overall facilitation. Sometimes our debriefs focused on lament; for example, we shared disappointment in ourselves for freezing in moments we believed we should have intervened. Other times, we discussed the impacts of our facilitation on each other, and our needs from each other in terms of when to intervene and/or step back. Our shared commitments to antiracism were imperfect but insistent, and kept us grounded in moments of uncertainty.

## Critical race consciousness: A requisite for appreciation

Our collective reflexive process led us to contend that researchers’ ability to fully appreciate the positive core, “the best of what is” of an organization (Fifolt & Stowe, 2011), necessitates understanding how race influences both data interpretations and the overall research process. We conceptualize this understanding as critical racial consciousness (Carter, 2008). Examining our own and our participants’ social locations is one component of critical racial consciousness, and this process was crucial to our appreciation of the organization.

We came together through our shared commitment to antiracism research and pedagogies, but we approach our work from different social locations, as a first-generation Filipina, a white woman of Eastern European descent, and a white Ashkenazi Jewish settler woman. Our subjectivities are formed by long lineages that were always in the room with us, shaping our research; For Pino, her migration to Canada from the Philippines was impacted by the dominant narrative of Canada as a ‘greener pasture’ that can offer a better life than one’s home country. This dominant narrative is a myth as many Filipinos in Canada continue to live below the poverty line even after retirement due to experiences of racism and de-professionalization during their working years (Coloma & Pino, 2016). As a trans woman of colour who also has worked in the settlement sector, Pino observed how the same narrative popped up among research participants, validating our shared desire to make explicit an antiracist approach for Ai to question these racial and nationalistic discourses. For Gebhard, her ancestors’ white skin granted them automatic entitlement to the stolen resources of the nation; however, she is most familiar with oppressive narratives of meritocracy that present inequality as a problem of individuals’ lack of work ethic. Her experiences teaching in the school system provoked her reflection on (including about her own complicity in) the white settler resentment of newcomers of colour whose presence challenges white dominance. For Allen, her entry into this work is informed by her familial trajectory and life of “diasporic sensibilities” (Walcott, 2003), shaped by her partner’s experiences as an African immigrant in Canada; by her work that variably attends to the entanglements of settlement and settler colonialism; and by the tracings of white women’s (including her own) implication in settlement, education and social sectors of the state (Allen, 2022).

Elaborating on the above, we folded into our focus group introductions how our commitments to antiracism have come to be. Since racialized people can face social penalties for naming race and racism, we believe these acknowledgments were crucial to support participants to share more openly. While we surmise this was useful for opening space for counterstories in our focus groups, we also recognized broader expectations of positivity from participants. Antiracist efforts are often thwarted by the assumption that conversations must focus on “making whites happy or at least feeling positive about being white” (Ahmed, 2012, p. 169), and our missteps reminded us we are not immune to these assumptions.

We hope our intentionality around not feigning neutrality may have supported participants’ discussions of race and racism, and also disrupted the normative expectations that participants may have associated with our social locations. While the three of us are impacted differently by discourses of neoliberal multiculturalism outlined earlier, we are familiar with the scripts these ideologies expect of our particular social locations. The scripts that signal valorized ways of enacting white femininity and performing normative stereotypical Filipina identity in our research context include the superficial celebration of ethnic identity, obscuration of white supremacy, denial of racism, and claims of colour-blindness.

We were thus attentive to how such scripts played out in the research process. For Allen and Gebhard, this meant being intentional about not reinscribing the historical scripts of white settler femininity, including colonial impulses of ‘benevolence’ and performances of self-appointed expertise on racialized people’s lives and experiences (Allen, 2022). For Pino, racialized participants’ narrations on how they support racialized newcomers and refugees during COVID-19 reminded her of her own lived experiences of racism, and her community work with Filipino migrants and social service providers. The discourse of neoliberal multiculturalism has oftentimes positioned her community organizing efforts as her natural responsibility rather than the responsibility of the nation-state. A salient insight for Gebhard was the recognition of her desire to hear positive and “happy” stories about settlement in Canada, and she grappled with how this influenced her comportment with participants and her reading of the data. Second, Gebhard felt compelled at certain points to soothe participants who had disclosed stories about racism, and she reflected on how such gestures can be subordinating. For Allen, returning to the transcripts became a re-witnessing of how “Whitespeak” (Moon, 1999) can operate in pernicious ways through distanced or disembodied forms of antiracist discourse, and how this can demand the heaviest labour of racialized colleagues (Dhamoon, 2020). To re-witness herself as a researcher long after data collection offered her accountable opportunities to re-read in order not to reproduce white power-laden communicative practices of speaking too much when it is time to be quiet, and staying silent when it is time to challenge harmful discourses.

Another component of critical race consciousness is the identification of how neoliberal multicultural discourses can operate within Ai’s positive frame, as they are constructed as celebratory, inclusive, and offer contained solutions (e.g., cultural sensitivity training) and not systemic approaches, as antiracism requires (Dei & Johal, 2005; Fleras, 2014). We grappled with how to navigate the silencing effects of neoliberal multicultural discourses, particularly when they were stated by white participants, and how we may have better intervened in order to (re)center counter discourses.

The above reflection was provoked by one particular focus group that consisted of a white settler male, two white settler females, and one racialized woman who had recently started in her settlement support role. We acknowledged the exacerbation of discrimination, racism and xenophobia during the pandemic, and inquired about participants’ associated experiences in their work. The space became occupied by deflective discourses of neoliberal multiculturalism, voiced and backed up by white participants. Focus and power shifted to (re)center white masculinities, for example in terms of dominating talk time; and white femininities, for example in terms of reifying the benevolence of white women educators. White participants made statements about the ‘goodness’ of teachers and minimized the impacts of discrimination. And, the white male participant spoke in what we interpreted as an authoritative tone about race being a social construct; while this is fitting with our own framework, we were interested in understanding the impacts this construct has for racialized students. While we cannot speak to the intention of these statements, we surmise they impacted the group’s ability to speak about racism, for fear of contradicting the white male’s stance. In the same group, we witnessed the popular maneuver of locating the solution to racism in “talking about different cultures,” through suggestions of activities that celebrate difference but fail to consider unequal power relations, thereby rendering everyone as the same or equal: “Let’s celebrate human– it’s not any specific life that matters. Every life matters. Yeah, so we can celebrate humanity…” This quote further demonstrated for us the impact of the white male’s statements, which–while perhaps unintendedly–seemed to give permission to other group members to deny the salience of race and racism for newcomer students in schools.

We probed the white participants to re-center the material impacts of racism. Referencing her work with Filipino youth in Toronto, Pino noted “those [opportunities for discussing race] have allowed us to really recognize the significance of race, and how it impacts the lived experiences of people…they see it in their everyday lives, from their parents, from their families who have been impacted by racism in unemployment, and their lack of recognition of their parents’ skills and professional experience from the Philippines.” In another instance, noting participants’ usage of coded language including “they” and “those students”, Allen asked participants to use newcomer students’ names (pseudonym) or racial identities. To underline the relationship of racial categories and educational opportunities, Gebhard stated: “Newcomer youth or youth of colour are more often targeted for discipline, or their behaviour is perceived differently than that of white students, what you are referring to today as ‘mainstream’ students.”

In spite of the above examples, we believe there was limited space and safety for the racialized woman participant to share counterstories. Towards the end of the focus group, she used the word racism to describe a client’s experience at work then quickly and repeatedly apologized, underlining she did not imply “*all* Canadians.” We responded by reaffirming that no apology for naming racism was necessary, and expressed appreciation for her sharing. We continue to reflect on how the focus group dynamics of whiteness may have made an apology feel necessary, and how her and other racialized participants may have felt they needed to protect themselves in the focus groups. Indeed, racialized newcomers can face cultural and social reprisal for refusing to perform the expected ‘grateful', ‘good', ‘obedient' immigrant employees, or ‘proper' citizen-subject of the nation-state. We reflected together about the unfairness of assuming focus groups are devoid of the oppressive power relations racialized people navigate in other spaces, and the complexities involved.

As illustrated, liberal multicultural discourses within Ai can work to silence participants’ articulations that may be perceived as ‘negative’, reproducing oppressive and sanitized interactions. Such relational conditions, and the power inequalities they maintain, can be compounded by the social locations of the facilitators and research participants. Learning from the above experience, we were subsequently more explicit in our foregrounding of antiracism and its distinctions from neoliberal multicultural discourses in our introductions. Moreover, we considered how we might intentionally use racial caucus groups in the future as part of an antiracist Ai process, which would mean grouping participants according to racial identities. Research suggests this strategy can mitigate the reproduction of racial hierarchies in antiracist education (Halvorsen et al., 2022), and we contend it could serve a similar purpose in focus groups.

## The limits of happiness: A requisite for revealing the positive core

As a hallmark of Ai, participants are asked to identify the positive core of the organization, which is the foundation through which the Ai stages are articulated. An ongoing theme throughout our research meetings related to the value-laden nature of positivity; specifically, we reflected on how neoliberal institutional framings of positivity could lead to silencing participants’ stories of the pandemic’s impacts and experiences of discrimination. Our reflections emerged from what Hynes (2013) might describe as “first person inquiry’s mindful engagement with…power, different worldviews, and unquestioned truths” (p. 54).

Tensions emerged as we grappled with placing the expectations of positivity upon a group of predominantly racialized contract workers when conditions of personal and professional uncertainty, were evoking a range of emotions. While the participants would ideally shape the meaning of positivity for themselves, we wanted to be responsive to institutional contexts where demands of positivity and of happiness for newcomer and immigrant people is a disciplinary technique, materialized by expectations of gratitude from newcomers and articulated in comments such as: “How ‘lucky’ you are to be here and to have this job!” (Ahmed, 2012; Tungohan, 2012). Moreover, this disciplinary technique is an institutional investment in diversity branding that positions the institution as inclusive and equitable, evidenced by the happy, smiling worker (Liu, 2020). The demand of happiness and positivity can also operate as a threat within conditions of employment precariousness and insecurity, as well as within conditions of risk to health and safety, as exemplified during the pandemic. Seen to curtail workers’ productivity, and in turn capital accumulation, the ‘unhappy’ or ‘negative’ worker does not align with the institution’s competitive advantage in the neoliberal market, and racialized workers are required to posture positivity and happiness despite their emotional predicaments (Pino, 2014).

In our study, the participants, the participants’ clients, and most members of the study’s research advisory committee were situated in institutions that expect happy, thankful migrant subjects (Allen et al., 2021). The effects of what Ahmed (2012) calls the “happiness duty” (p. 156) were articulated at various points in our research process. For example, we learned how settlement workers in schools felt compelled to respond positively, or to ignore, the patterns of institutional inequities such as micro-aggressions they witnessed in their work, to prioritize good relations with white teachers and staff. When we presented a particularly egregious example of racism that one participant witnessed in their school to our research advisory committee, uncertainty was expressed about the veracity of the story. Racism was also presented in the same meeting as something school settlement workers could avoid, through hard work and a pleasant demeanour. Recognizing the need for a nuanced interpretation of these examples, we speculate that a desire not to name racism may be a means of institutional survival, and a form of protection to manage the risks to safety and program continuity. Due to requests from our research advisory committee and our recognition of the real social penalties the organizations could face as a result of what we wrote, we softened our language and removed a theme about racism.

The above reflections led us to grapple how counter-stories that might be perceived as “negative,” were a key pathway to the organization’s positive core. We reframed positivity and by implication we reframed negativity; we emphasized that telling stories of racism is not inherently negative. By opening up to a plurality of stories and counterstories, we conceptualized stories as forms of resistance and regeneration, which allowed us to witness participants’ agency and to reimagine what constitutes positivity from an antiracist frame. The potential for empowerment through an opportunity to express emotions freely was demonstrated in our research through several participants’ comments about the affirmation they felt from the opportunity to share their stories. This antiracist reimagining supports a plural ‘and/with’ relation and a move away from a singular positive/negative binary (Bushe, 2012). By extension, a richer range of emotions beyond happiness can be expressed. Such emotions include anger and rage, which as Black scholars, Indigenous scholars, and scholars of colour have powerfully illustrated, are indeed empowering and strengthening sources of love, life, activism, and regeneration (Arvin et al., 2013; Johal, 2005).

## Recognition of transnational knowledges: A requisite for inquiry

Our practice of appreciation included our recognition of participants’ transnational knowledges and lived experiences of settlement. Transnational knowledge pertains to the multiple ontologies *beyond* the Western nation-state, and this purposeful shift in orientation was in keeping with the fundamental aspect of first person inquiry that asks researchers to question taken for granted assumptions about the world (Hynes, 2013). We re-conceptualized ‘the positive’ as participants’ strength, support, and commitment to newcomer students and their families, and as their visionary animations of breaking down barriers and inequalities in educational systems so newcomer students can thrive. This re-conceptualization connects critical appreciative inquiry to antiracism in recognizing people have multiple ways of knowing, and that engagements in resistance are strength-based, life-giving acts that create positive *counter*-narratives of agency and change. Transnational knowledges and expertise are often dismissed and devalued through lack of credential recognition and other institutional barriers, as thematically articulated by participants. However, their stories illustrated the significance of their transnational knowledges and expertise for meaningfully connecting with newcomer students and families. For example, one participant spoke about her diligent efforts to ensure a student was not streamed into a special education program; the participant recognized the student’s struggles were rooted in inadequate English language support, *not* in cognitive ability, as the special education referral implied. We also learned how crucial settlement workers’ multilingual skills were during the pandemic, at a time when communicating to newcomer families was crucial to their survival and well-being. Pathologizing stories about newcomer students and families that emphasize deficits abound, and the ability to tell alternative and disruptive narratives emanates from participants’ transnational knowledges.

The appreciation for transnational knowledges was expressed within the focus groups. For example, during the discovery phase, participants spoke about the high value placed on technologically mediated communication in Canada, and assumptions newcomer students and families would have equal access to and shared valuation of technology. Participants identified how this translated into barriers for newcomer students. One participant noted “a big issue all families encounter when they come to Canada, is the value that…Canadian culture places on technology versus the value that in other countries…we place more value in face to face interactions…there’s a lack of empathy from the Canadian culture regarding the value that they place in technology versus the face to face interaction.”

In response, Pino, drawing on her transnational experience, validated participants’ articulations through her shared understanding of the impact of western valuations of technology and constructions of time. She stated, “You’re really raising a very good point and the way in which cultures plays a big role in terms of how communities or families look at technology…it’s also a matter of how culturally we imagine time, because I can totally relate to you, and coming from a very collectivist culture, sometimes we don’t need technology, we need face to face contact and we also have very different work dynamics with professionals.” By recognizing collectivist ways of relating to each other and to time, Pino denaturalized the dominant technological and temporal norms of the participants’ institutions in Canada. This act can also be read as one of shifting power and authority away from white Western Euro-Canadian temporal practices. The appreciation of transnational knowledge opened ontological space in the focus group for participants in the discovery and dream phases to consider change beyond assimilation into the architecture of current systems. In other words, instead of changing individuals, explicitly opening space for the participants’ transnational knowledges enabled the conversation to shift towards how systems, practices and norms of whiteness need to change instead.

## Final reflections and conclusions

Through our practice of collaborative reflexivity, we have narrated how we followed an antiracist epistemology to negotiate the tensions we felt with the neo-liberal discourses of Ai. Without a theoretical framework that attends to racism and power, Ai may not capture research participants’ transnational knowledges; the work realities that forestall transformation; nor the full range of emotionality, experiences, and counternarratives that exist outside the confines of a positive/negative binary. A *commitment* to honoring an organization’s positive core is alone insufficient; a critical race consciousness deepens the possibility that a fulsome appreciation of an organization’s strengths will be accomplished. Since Ai is an action-oriented methodology that aims to foreground the expertise of participants, a lack of attention paid to power relations risks generating findings that falsely locate “problems” within minoritized groups, thereby contradicting Ai’s larger goals of appreciation and amplification of marginalized people’s voices.

Reorienting along antiracist lines is not to abandon notions of appreciation or positivity; instead such reorientation can deepen structural analyses and enrich reflexivity. We align with scholars such as Bushe (2012) who argue for a shift towards generative subjects instead of positive subjects, and Grant and Humphries (2006), who recognize that “critique, however, need not equate with criticism and negativity” (p. 408).

To Dream in Ai is to go “beyond the status quo and formulating visions and documenting future scenarios” (Bergmark & Kostenius, 2018, p. 624). An antiracist approach allowed us to recognize what limits dreaming and design; in our case, we identified limitations imposed by neoliberal institutional discourses within the context of a global pandemic. We found that the participants’ dreaming and designing ideas were principally about dignity, equity and justice for newcomer students and families.

By reflecting back, we also cast forward, to consider what we will do differently in another antiracist articulation of Ai. Accountability in our research process means being equally reflexive about how we have narrated this process herein, and the tensions and failures left unresolved. As part of antiracist practice, we remain committed to further cultivating new skills and literacies to enrich our Ai practice. We continue to question how researcher accountability can be deepened throughout each research stage. Our hope is that this article will inspire other researchers to mobilize critical Ai, to increase the likelihood that the methodology will genuinely serve the interests of participants by direct action and social advocacy.

## Notes

1. Newcomer is a term sometimes used interchangeably with recent immigrants; newcomer refers to landed immigrants who came to Canada up to five years prior to a given census year (Statistics Canada, 2016).

2. The term “Indigenous peoples” used to refer to First Nations, Métis, and Inuit people. First Nations people are original inhabitants of the land and the self-ascribed names of the First Nations of Saskatchewan are: Nêhiyawak^1^ (Plains Cree), Nahkawininiwak (Saulteaux), Nakota (Assiniboine), Dakota and Lakota (Sioux), and Denesuline (Dene/Chipewyan) (Stonechild, n.d.). Originally a term for people of First Nations and European ancestry, Métis Nation Saskatchewan defines Métis as “a person who self identifies as Métis, is of historic Métis Nation ancestry, is distinct from other Aboriginal peoples, and is accepted by the Métis Nation (2022)” (Métis Nation Saskatchewan; also see Vowel, 2016). Inuit refers to Arctic Indigenous peoples, the majority of whom inhabit the northern regions of Canada.

3. Recognizing the imperfect collapsing and essentializing risks of identification terms, we turn to Vowel (2016)’s definition of the term settler to reference the original European settlers, who “brought with them their laws and customs, which they then applied to Indigenous peoples and later to all peoples who have come to Canada...” as well as to “people who continue to move into Canada and settle here…” This is with the *explicit* recognition that “non-European peoples are folded into the settlement process when they arrive here — even as they are often denied equal social privileges [and] do not have the power to bring with them their laws and customs” (pp. 16–17; also see Chatterjee, 2019; Jafri, 2012).
